# Correction: A screen of drug-like molecules identifies chemically diverse electron transport chain inhibitors in apicomplexan parasites

**DOI:** 10.1371/journal.ppat.1011987

**Published:** 2024-02-01

**Authors:** Jenni A. Hayward, F. Victor Makota, Daniela Cihalova, Rachel A. Leonard, Esther Rajendran, Soraya M. Zwahlen, Laura Shuttleworth, Ursula Wiedemann, Christina Spry, Kevin J. Saliba, Alexander G. Maier, Giel G. van Dooren

In the Abstract, one of the compounds referenced is incorrectly named MMV024937. The correct compound is MMV024397.

Also, in Fig 9, the x axis of panel H is incorrectly labelled as MMV024937. The authors have provided a corrected version here.

**Fig 9:**
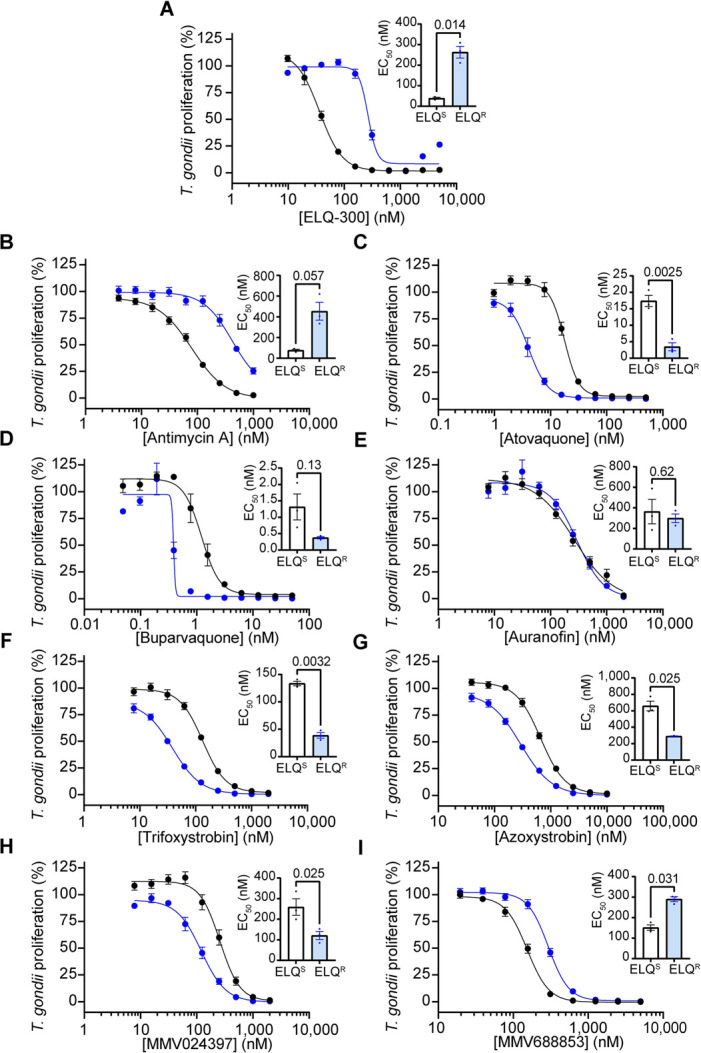
Assessing the activity of ETC inhibitors against ELQ-300-resistant *T*. *gondii* parasites. (A-I) Dose-response curves depicting the percent proliferation of ELQ-300-resistant (ELQ^R^, blue) *T*. *gondii* parasites, or the corresponding ELQ-300-sensitive parental strain (ELQ^S^, black), in the presence of increasing concentrations of (A) ELQ-300, (B) antimycin A, (C) atovaquone, (D) buparvaquone, (E) auranofin, (F) trifloxystrobin, (G) azoxystrobin, (H) MMV024397, or (I) MMV688853. Values are expressed as a percent of the average fluorescence from a no-drug control at mid-log phase growth in the fluorescence proliferation assay, and represent the mean ± SEM of three independent experiments performed in triplicate; error bars that are not visible are smaller than the symbol. Inset bar graphs depict the EC_50_ ± SEM (nM) of three independent experiments. Paired t-tests were performed and *p*-values are shown.
