# Supplementary figures and images for: A screen of drug-like molecules identifies chemically diverse electron transport chain inhibitors in apicomplexan parasites
Source: PLoS Pathog. 2023 Jul 20;19(7):e1011517. doi: 10.1371/journal.ppat.1011517 (PMC10403144; doi:10.1371/journal.ppat.1011517)

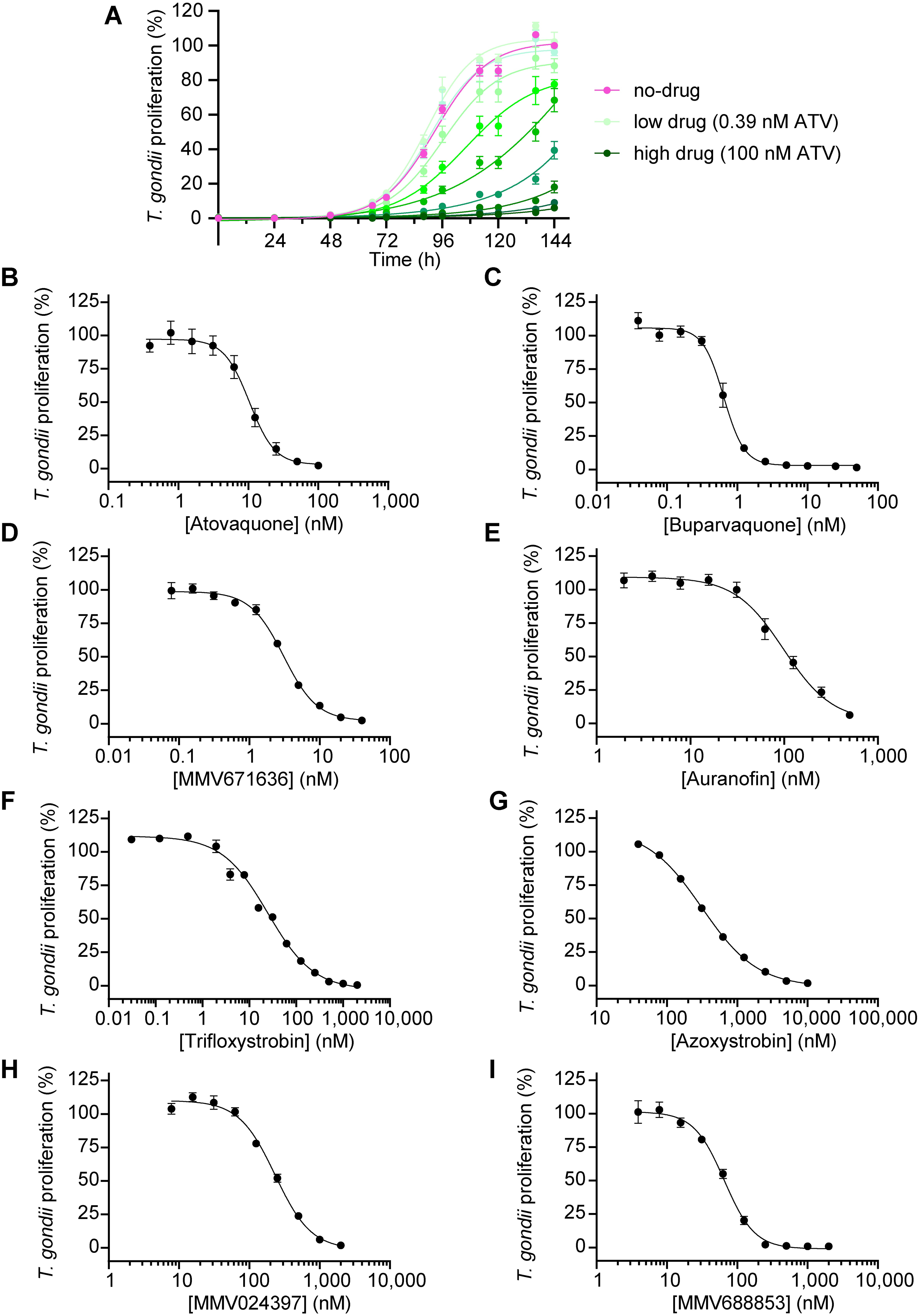

Supplement: S1 Fig — (A) Proliferation of tdTomato-expressing T. gondii parasites cultured in the absence of drug (pink circles), or in the presence of atovaquone (two fold serial dilution from highest concentration (100 nM; dark green) to lowest concentration (0.39 nM; light green)) over a 6-day period. Values are expressed as a percent of the average fluorescence from the no-drug control on the final day of the experiment, and represent the mean ± SD of three technical replicates. Data are from one experiment and are representative of three independent experiments. Similar proliferation curves were obtained for each test compound. (B-I). Dose-response curves depicting the percent of T. gondii parasite proliferation in the presence of a range of concentrations of (B) atovaquone, (C) buparvaquone, (D) MMV671636, (E) auranofin, (F) trifloxystrobin, (G) azoxystrobin, (H) MMV024397, or (I) MMV688853. Values are expressed as a percent of the average fluorescence from the no-drug control at mid-log phase growth, and represent the mean ± SEM of three independent experiments, each conducted in triplicate; error bars that are not visible are smaller than the symbol. (TIF) [file ppat.1011517.s002.tif]

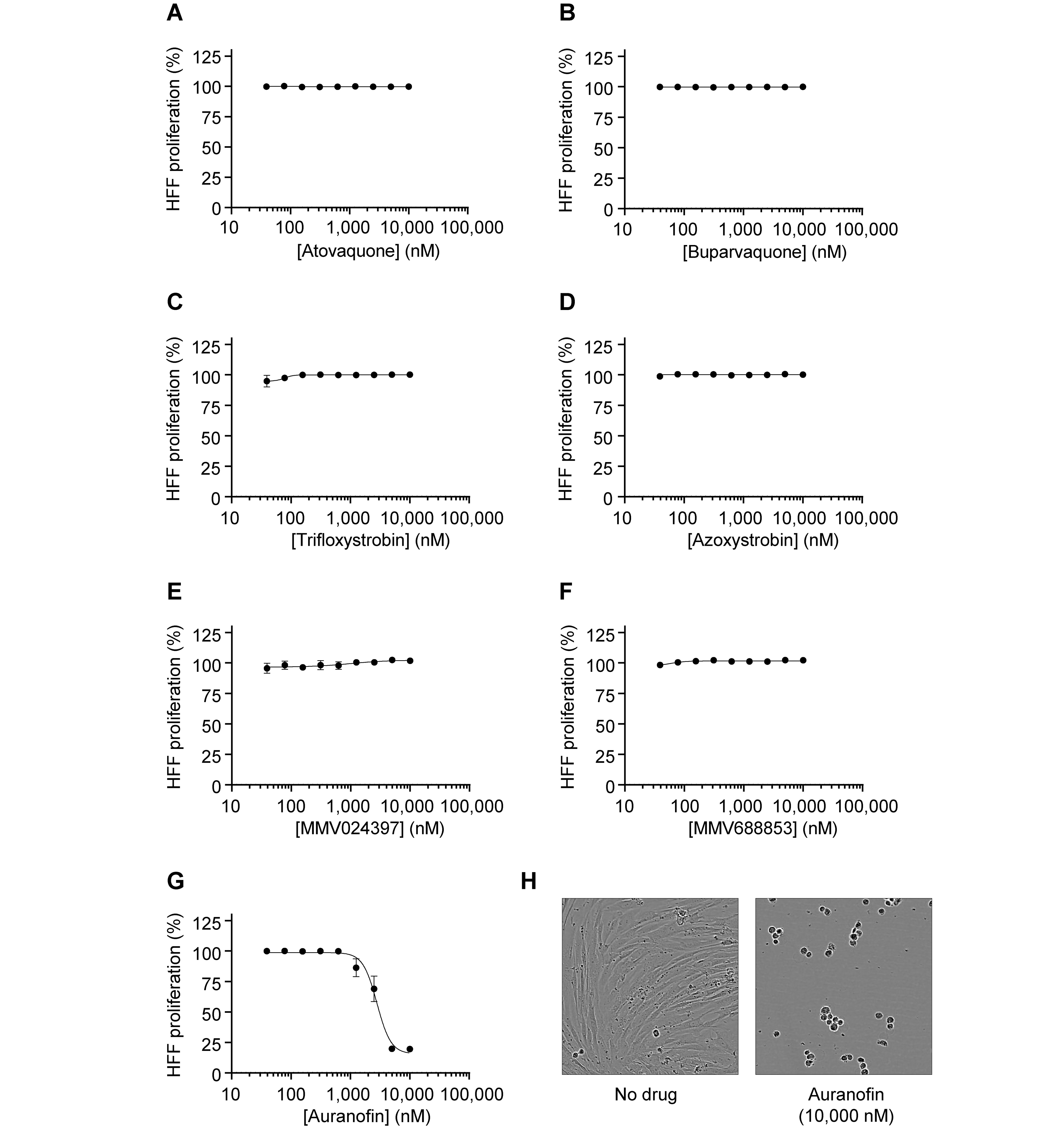

Supplement: S2 Fig — (A-G). Dose-response curves depicting the proliferation of human foreskin fibroblast (HFF) cells in the presence of a range of concentrations of (A) atovaquone, (B) buparvaquone, (C) trifloxystrobin, (D) azoxystrobin, (E) MMV024397, (F) MMV688853, or (G) auranofin. Values are expressed as a percent of the average confluence of the no-drug control at the end point of the assay (after 4 days of proliferation), and represent the mean ± SEM of three independent experiments, each conducted in triplicate; error bars that are not visible are smaller than the symbol. (H) Representative images of HFF cells at the end point of the assay when grown in the absence of drug (left; no drug) or in the presence of 10,000 nM auranofin (right). (TIF) [file ppat.1011517.s003.tif]

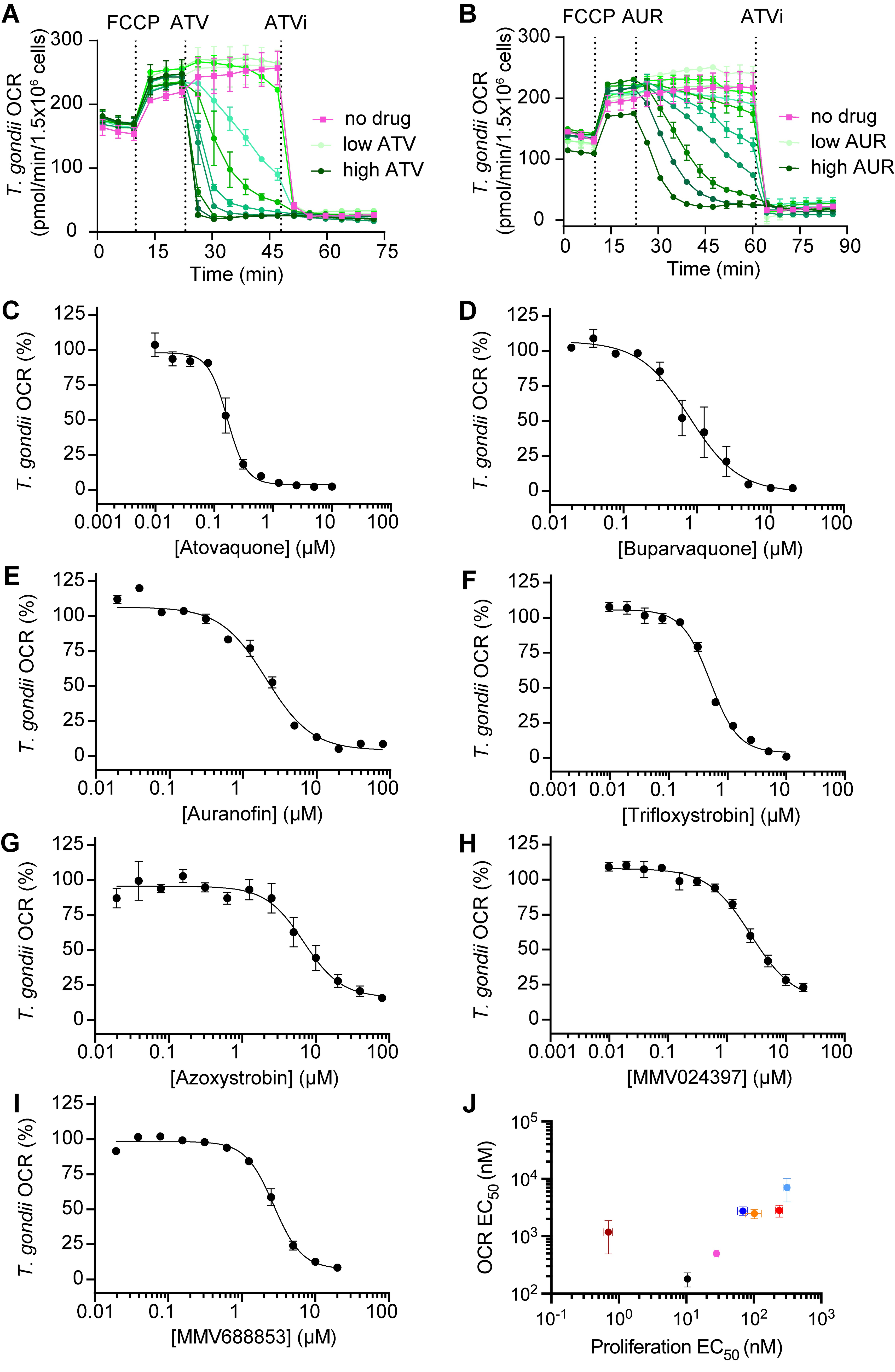

Supplement: S3 Fig — (A-B) Traces depicting the changes in O2 consumption rate (OCR) over time of intact T. gondii parasites incubated with no drug (pink) or with (A) atovaquone (ATV; two fold serial dilution from highest concentration—10 μM, colored dark green—to lowest concentration—0.01 μM, colored light green) or (B) auranofin (AUR; two fold serial dilution from highest concentration—80 μM, colored dark green—to lowest concentration—0.08 μM, colored light green)). FCCP (1 μM) was injected into the well to uncouple electron transport from the proton gradient and thus elicit the maximal OCR. A range of concentrations of the test compounds were then injected and the inhibition of OCR measured over time. A final injection of an inhibitory concentration of atovaquone (ATVi; 5 μM) maximally inhibited mitochondrial OCR. Values represent the mean ± SD of two technical replicates from a single experiment and are representative of three independent experiments. Similar OCR inhibition traces were obtained for each test compound. (C-I) Dose-response curves depicting the percent of T. gondii OCR in the presence of increasing concentrations of (C) atovaquone, (D) buparvaquone, (E) auranofin, (F) trifloxystrobin, (G) azoxystrobin, (H) MMV024397 or (I) MMV688853. Values represent the percent OCR relative to the no-drug (100% OCR) and inhibitory atovaquone-treated (0% OCR) controls, and depict the mean ± SEM of three independent experiments, each conducted in duplicate; error bars that are not visible are smaller than the symbol. (J) Comparison of the EC50 values determined for T. gondii OCR (OCR EC50; this figure and Table 3) and WT T. gondii proliferation (proliferation EC50; S1 Fig and Table 1). Coloring of compounds is as in Figs 1 and 3 (atovaquone, black; buparvaquone, burgundy; auranofin, orange; trifloxystrobin, pink; azoxystrobin, light blue; MMV024397, red; and MMV688853, dark blue). (TIF) [file ppat.1011517.s004.tif]

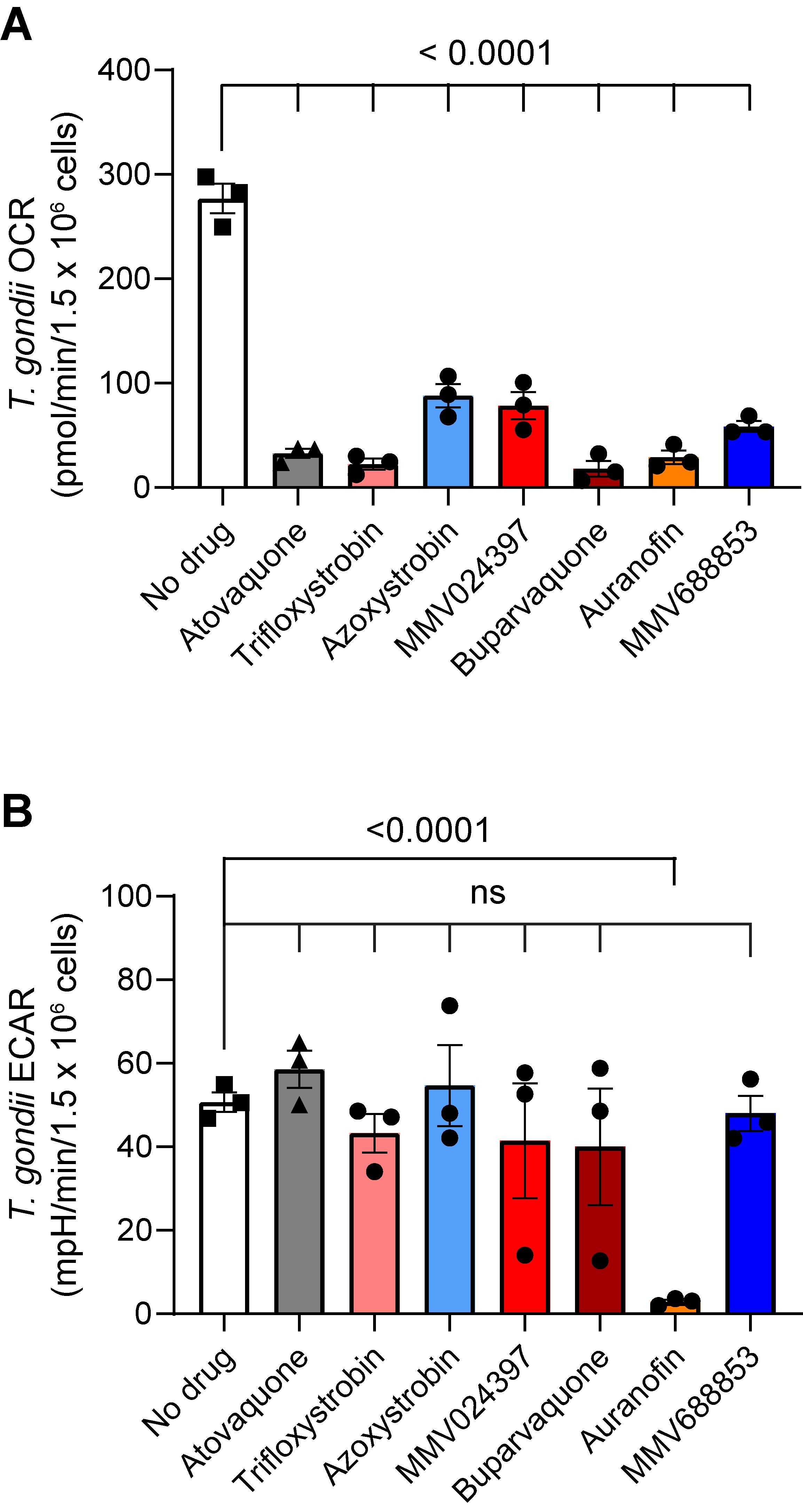

Supplement: S4 Fig — (A) O2 consumption rate (OCR) and (B) extracellular acidification rate (ECAR) of T. gondii parasites treated with either no drug (white), atovaquone (gray; 10 μM), trifloxystrobin (pink; 10 μM), azoxystrobin (light blue; 80 μM), MMV024397 (red; 20 μM), buparvaquone (burgundy; 20 μM), auranofin (orange; 80 μM) or MMV688853 (dark blue; 20 μM), assessed using a Seahorse XFe96 flux analyzer. Bars represent the mean ± SEM of three independent experiments each conducted in duplicate. ANOVA followed by Dunnett’s multiple comparisons test were performed and p-values are shown. A graphical output of these data comparing OCR to ECAR is depicted in Fig 3A. (TIF) [file ppat.1011517.s005.tif]

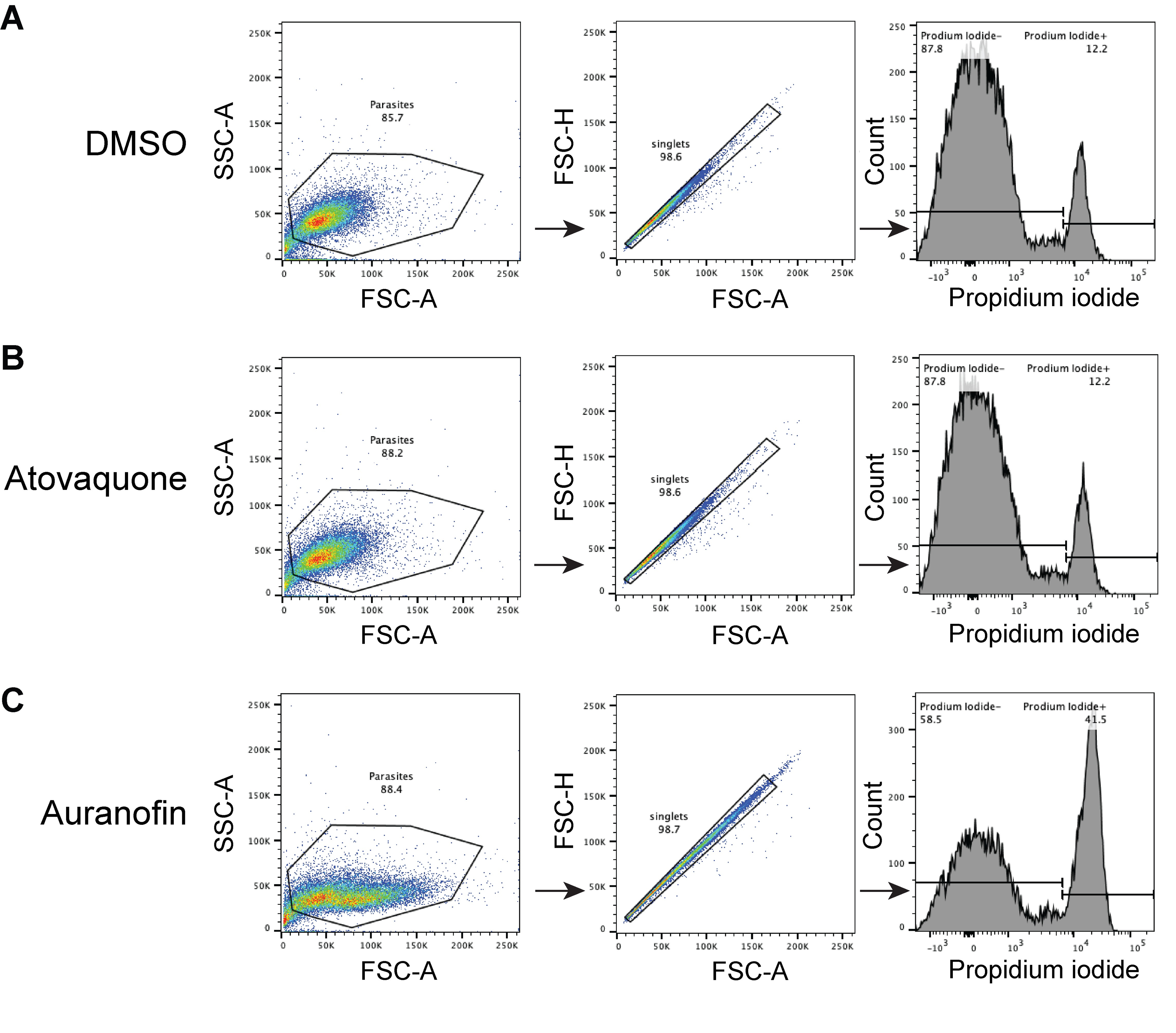

Supplement: S5 Fig — (A-C) Extracellular parasites treated with (A) DMSO, (B) atovaquone, or (C) auranofin were gated away from debris using SSC-A and FSC-A parameters (left plots). Single parasites were subsequently gated using FSC-H and FSC-A parameters (centre plots). Propidium Iodide (PI) fluorescence (right plots) was used to separate viable parasites (PI negative) from non-viable parasites (PI positive). Graphs depict data from the DMSO, 10 μM atovaquone and 20 μM auranofin treatments at the 80 minute time point from a single experiment, and are representative of the gating strategy used in three independent experiments at the compound concentrations and treatment times listed in Fig 3B. (TIF) [file ppat.1011517.s006.tif]

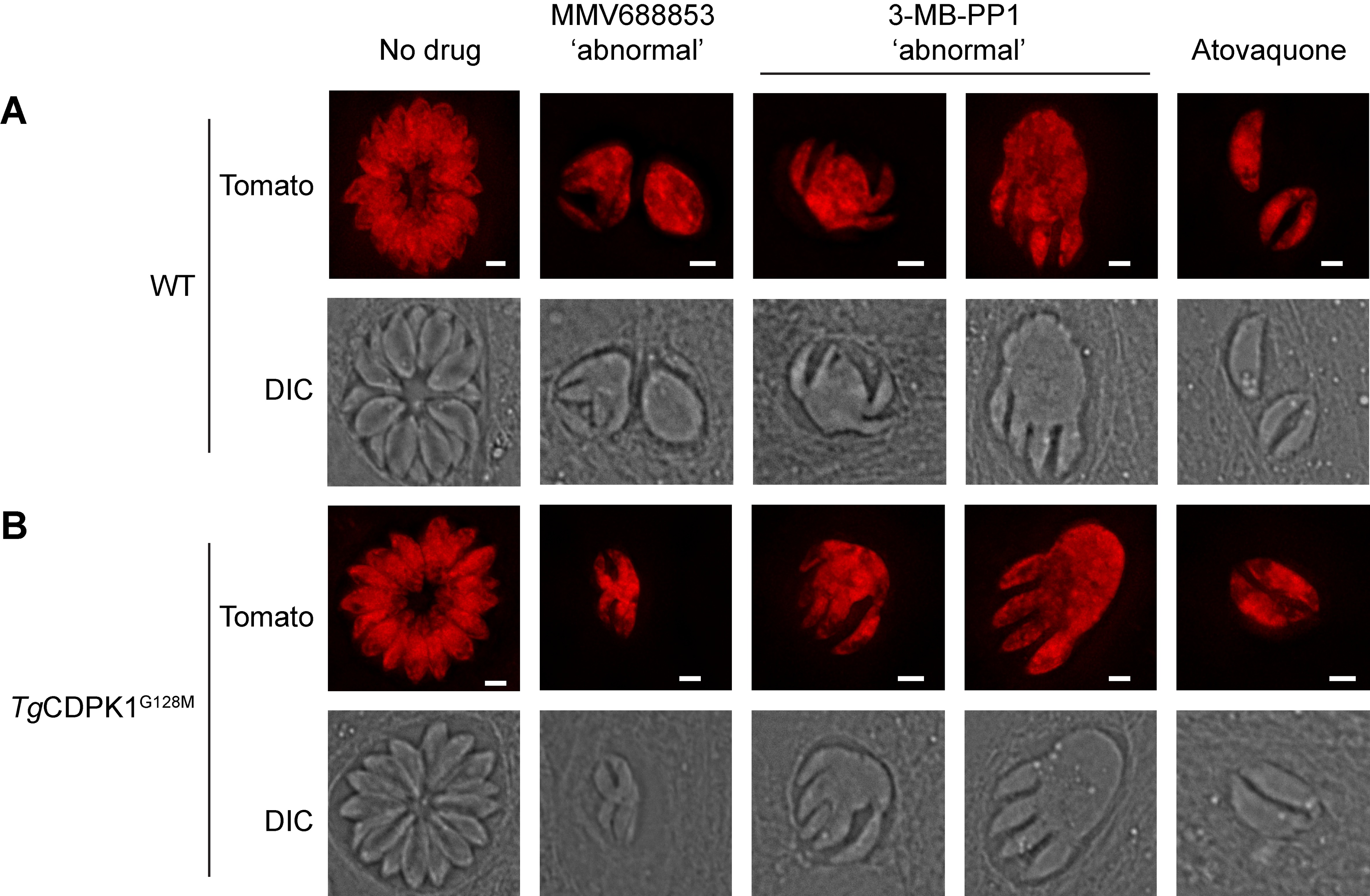

Supplement: S6 Fig — Representative images of (A) WT or (B) TgCDPK1G128M parasites expressing tdTomato observed during intracellular proliferation assays, with tdTomato fluorescence (top) and differential interference contrast (DIC; bottom) images depicted. WT or TgCDPK1G128M parasites were cultured in the absence of drug (no drug), or the presence of MMV688853 (5 μM), 3-MB-PP1 (5 μM) or atovaquone (1 μM) for 20 h. Abnormal morphology was defined as vacuoles that contained misshapen parasites as depicted in the images of MMV688853 and 3-MB-PP1 treated parasites. Scale bars are 2 μm. (TIF) [file ppat.1011517.s007.tif]

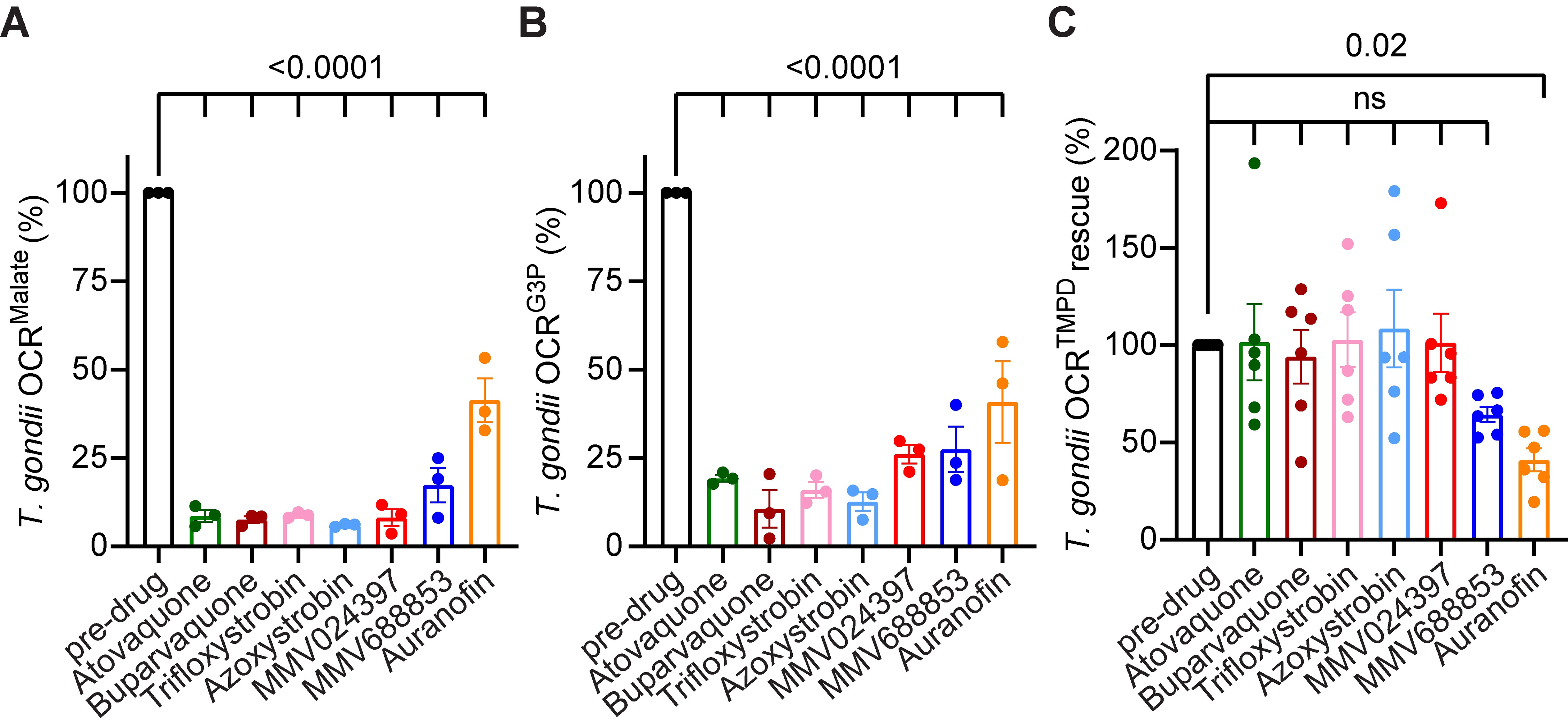

Supplement: S7 Fig — Quantification of the change in the (A) malate- or (B) glycerol 3-phosphate-dependent O2 consumption rate (OCR) of plasma membrane-permeabilized T. gondii parasites after injection of inhibitors (atovaquone, 1.25 μM; buparvaquone, 5 μM; trifloxystrobin, 2.5 μM; azoxystrobin, 80 μM; MMV024397, 20 μM; MMV688853, 20 μM; auranofin, 10 μM). (C) Quantification of the rescue of OCR by TMPD after inhibition by the above compounds. Data were normalized relative to the baseline OCR level pre-substrate injection (0% OCR) and the malate/G3P-elicited OCR level pre-drug injection (100%). Data represent the mean ± SEM of three independent experiments each conducted in at least duplicate. Note that the TMPD graph combines data from both the malate and G3P experiments. Error bars that are not visible are smaller than the symbol. ANOVA followed by Dunnett’s multiple comparisons test were performed and p-values are shown. Representative traces from which these data were quantified are depicted in Fig 5. (TIF) [file ppat.1011517.s008.tif]

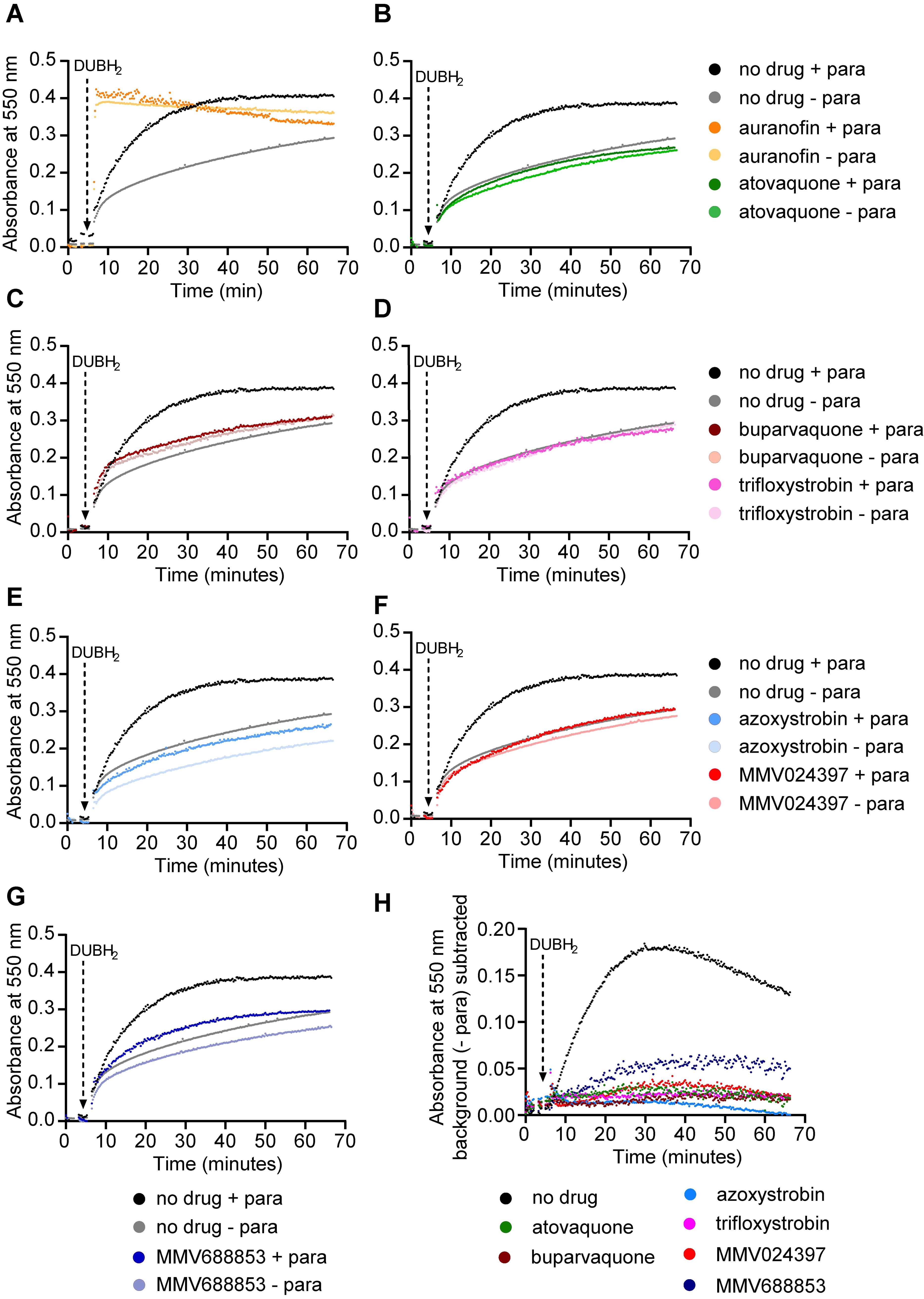

Supplement: S8 Fig — (A-G) Complex III activity assays showing the change in absorbance of equine heart CytC at 550 nm over time (measured every 15 s) in the presence (+ para, dark shade) or absence (- para, light shade) of parasite extracts, and in the absence of drug (no drug, DMSO vehicle control, black or gray), or in the presence of (A) auranofin (orange, 10 μM), (B) atovaquone (green, 1.25 μM), (C) buparvaquone (burgundy, 5 μM), (D) trifloxystrobin (pink, 2.5 μM), (E) azoxystrobin (light blue, 80 μM), (F) MMV024397 (red, 20 μM) or (G) MMV688853 (dark blue, 20 μM). Decylubiquinol (DUBH2) was added at the indicated times. Data are from a single experiment and are representative of three independent experiments. (H) Complex III activity assays showing the change in absorbance of equine heart CytC at 550 nm over time where change in absorbance in the absence of parasite extracts (i.e. background absorbance) was subtracted from the change in absorbance in the presence of parasite extracts. Data are from a single experiment and are representative of three independent experiments. (TIF) [file ppat.1011517.s009.tif]

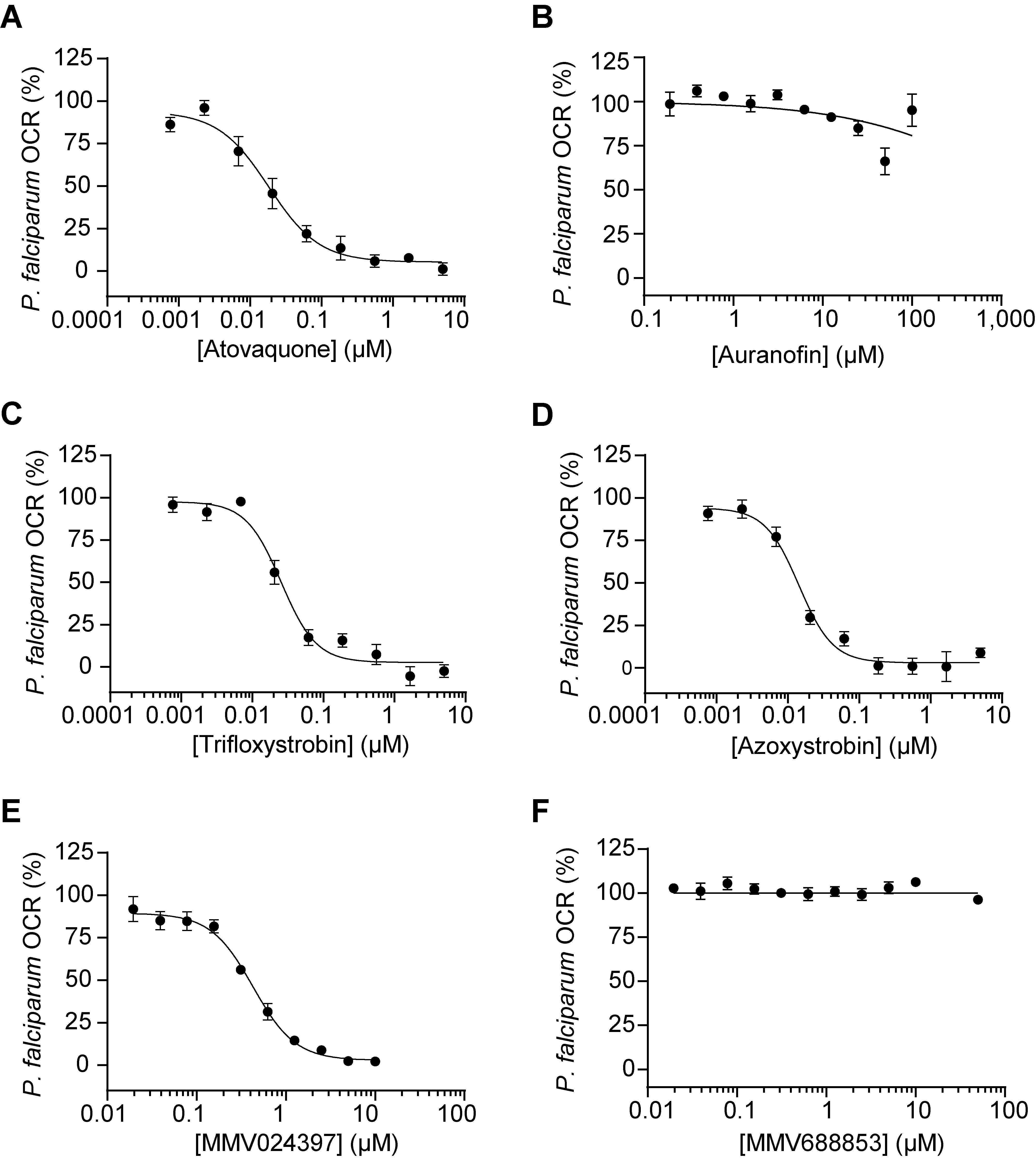

Supplement: S9 Fig — (A-F) Dose-response curves depicting the OCR of permeabilized P. falciparum parasites in the presence of increasing concentrations of (A) atovaquone, (B) auranofin, (C) trifloxystrobin, (D) azoxystrobin, (E) MMV024397 or (F) MMV688853. Values represent the percent OCR relative to the no-drug (100% OCR) and atovaquone-treated (0% OCR) controls, and are depicted as the mean ± SEM of three independent experiments; error bars that are not visible are smaller than the symbol. (TIF) [file ppat.1011517.s010.tif]
