# Supplementary material for: A screen of drug-like molecules identifies chemically diverse electron transport chain inhibitors in apicomplexan parasites
Source: PLoS Pathog. 2023 Jul 20;19(7):e1011517. doi: 10.1371/journal.ppat.1011517 (PMC10403144; doi:10.1371/journal.ppat.1011517)
Supplement: Attachment — Submitted filename: Response_to_Reviewers.pdf [file ppat.1011517.s011.pdf]

We have addressed the editor and reviewer comments through additional experimentation, additional data analyses, and changes to the manuscript text. We describe these responses in detail below (note that the reviewer comments have been edited to include only text that required a response; reviewer text is in black and our responses are in blue; we have numbered each editor/reviewer query to enable easier cross-referencing). We have tracked changes in a marked up manuscript (changes in red text).

Editor feedback: The reviewers raised a number of issues that will need to be addressed. Importantly, please

1. provide evidence for the robustness of the assay including correlation between growth inhibition and OCR inhibition and its scalability for HTP screening,

We now include a graph that compares the proliferation and OCR EC_{50} values of the inhibitors identified in our screen (S3J Fig). We find that there is a general correlation between EC_{50} values, with the most potent compounds against OCR also being the most potent proliferation inhibitors (with the exception of buparvaquone). We now note that EC_{50} values against OCR are higher across the board than against proliferation, possibly due to the different biological process being measured (e.g. 50% inhibition of OCR may lead to a proportionally greater effect on parasite proliferation) and the different time scales at which they are being measured (real time for OCR, several days for proliferation; Results, Lines 256-268). Regarding the robustness of the assay, we now report a Z' factor score for our assay, which is 0.3 for each plate (Methods, Lines 716-720). This suggests that we can separate hits from non-hits, although the Z-factor score is lower than ideal, thus necessitating the additional validation experiments that we conduct to characterise each candidate inhibitor. By scalability, we mean that we can screen larger compound libraries by screening more plates (Discussion, Lines 509-512). We have started screening larger compound libraries using the approach we describe in the manuscript, but these data are too preliminary to be included.

2. consolidate the work on MMV688853 ideally with the identification of the ETC target. However while SAR based on this molecule could help to characterize the basis of its dual activity, such investigation is clearly beyond the scope of this study.

We have undertaken additional experiments to further characterise MMV688853. We demonstrate that a parasite strain that is resistant to the Q_i-site inhibitor ELQ-300 is cross-resistant to MMV688853 (new Figures 9 and 10; Results, Lines 441-489). These data are consistent with the hypothesis that MMV688853 targets the Q_i site of Complex III. We agree that SAR studies of aminopyrozole carboxymides, and their abilities to target Complex III are now of considerable interest. We have commenced these studies, but they are still too preliminary to include in the present manuscript.

Reviewer #2 general comments:

3. While the authors are correct, the ETC is a validated target – the two major targets previously identified as DHODH and Cytochrome b. It is well known and well-documented that many small molecules identified in P. falciparum growth assay

screens target the ETC. In fact most groups do a counter-screen to identify ETC inhibitors, so the findings reported here are not surprising and are not novel. Others have screened the pathogen box and demonstrated several ETC inhibitors.

We are not aware of any published studies that have screened the Pathogen Box for ETC inhibitors. A 2019 study by Wang et al (PMID 31234346) screened the Pathogen Box for inhibitors of malate:quinone oxidoreductase (an enzyme that feeds electrons into the ETC) using a protein activity-based assay. Other screens of the Pathogen Box have identified some of the same compounds we identified in our screen as inhibitors of parasite proliferation, including in *Neospora* (Muller et al, 2017, PMID 28751177), *T. gondii* (Spalenka et al, 2018, PMID 29133550), *Babesia* and *Theileria* (Nugraha et al., 2019, PMID: 31254719 and Villares et al., 2022, PMID: 36380082). None of these studies tested for ETC inhibition or explored the molecular targets of the inhibitors experimentally. The activity of the compounds identified in our screen against multiple apicomplexan species suggests that the approach we are using can enable the identification of pan-apicomplexan inhibitors of the ETC. We are also not aware of any studies that have used a Seahorse XFe96-based screening approach in other organisms, although some studies use targeted Seahorse XFe96 assays (*i.e.* not screening approaches) to characterise the molecular targets of inhibitors identified in broader screens (*e.g.* Rufener et al., 2018, PMID: 30396011, who screened the Pathogen Box for inhibitors in *Echinococcus*). Our study, therefore, is novel because it is the first to apply a targeted screen for ETC inhibitors that also facilitates identification of the molecular target of inhibitors within the ETC. An additional novel aspect of our study is the identification of compounds not previously characterised as ETC inhibitors in other organisms (MMV024397 and MMV688853).We highlight the benefits of our screening approach at various points in the manuscript, including in the closing paragraphs of the Introduction (Lines 149-152) and Discussion (Lines 603-608).

4. The work with the Seahorse technology is of interest and is valuable for the biochemical characterization of specific inhibitors. This paper demonstrates the Seahorse technology works with these parasites, however, this was an expected result. Noteworthy would have been the opposite result.

That the Seahorse technology works with these parasites is not something we are reporting for the first time in this study. We have previously used Seahorse-based assays to characterise genetic mutants lacking ETC complex proteins (e.g. Seidi et al., 2018, PMID: 30204084 and numerous other papers). More recently, we have developed Seahorse-based approaches that enable identification of where in the ETC impairment is occurring (Hayward et al., 2021, PMID: 33524071). These studies provide important genetic validation of our screening approaches and strategies. In the present manuscript, we build on this work and advance the field by applying this technology to drug screening (something that has not been done before). We have clarified all this in the opening to the final paragraph of the introduction (Lines 130-135).

5. The one set of novel experiments is the work with MMV688853. The Seahorse results provided validation that the compound inhibited ETC independent of its inhibition of TgCDPK1.

The authors go on to characterize the inhibitor MMV688853. This is by far the most interesting result in the paper and it is novel. This compound has previously been identified as a TgCDPK1 inhibitor and the work reported here strongly supports a dual action of this molecule as an ETC inhibitor in T. gondii. Interestingly MMV688853 has a different activity profile in P. falciparum. This points to differences in these systems – raising several biological questions. It also points to the potential for a dual inhibitor at least for T. gondii and SAR based on this molecule could help to characterize the basis of this dual activity.

See our response to query 2.

6. The work demonstrating activity of selected small molecues on atovaquone resistant parasites is interesting, but unfortunately not comprehensive. Resistance mutations in the cytochrome b gene have been demonstrated at several independent sites. Collateral sensitivity has also been observed with several small molecule pairs that target cytochrome b.

Our original manuscript tested a Q_o site mutant for cross-resistance to the compounds identified in our screen. We now include additional data that examines a Q_i site mutant that is resistant to ELQ-300 (McConnell et al, 2018 PMID: 30117728) for cross-resistance to the compounds from our screen. We observed that ELQ^R parasites remain sensitive to most of the inhibitors identified in our screen, and, curiously, ELQ^R parasites appear to be hypersensitive to many of the compounds (Fig 9 and additional data in Table 1). Our study therefore now incorporates both Q_o and Q_i site mutants. We agree with the reviewer's point that this is not a comprehensive analysis of resistance-conferring Q_o and Q_i site mutations – we pointed this out in our original manuscript and have added additional text to emphasise this further (changes underlined): "…we note that several other Q_o and Q_i site mutations can confer atovaquone or ELQ resistance [refs], and as such further, more comprehensive studies should test whether these compounds are effective against other ATV^R and ELQ^R strains. (Lines 554-557). The main purpose of screening studies such as ours is to identify candidate inhibitors for future characterisation and development. In our manuscript (which now comprises 3 data tables, 10 figures and 9 supplementary figures), we not only demonstrate that the screening approach we have developed can identify novel ETC inhibitors, we also begin to characterise their molecular targets in the ETC. Future studies that examine additional Q_o and Q_i site mutants, or which generate resistance mutations against the identified inhibitors to provide additional clues about their mode of action, are all very feasible, but would clearly constitute independent studies in their own right. Regarding the observation of increased sensitivity of the Q_o or Q_i site mutants to some of the identified compounds, we now reference a previous study that observed small increases in sensitivity of parasites containing an atovaquone-resistance conferring Q_o-site mutation to Q_i site inhibitors (Lines 550-552).

7. The focus and the title/abstract of the manuscript are misleading – this is at best a “mini” screen and on a set of compounds that has been previously screened by many. There is no evidence for the scalability and the idea of targeting the ETC is one well visited in the literature and by many previous screening campaigns.

We disagree that our title and abstract are misleading – we screen 400 compounds in a targeted assay on less than two full plates. Scaling this up is highly feasible. We comment on this in the discussion (Lines 509-512). Additionally, we reference other studies that undertake screens for ETC inhibitors (Introductions, Lines 113-125). As we point out in the manuscript, an advantage of our approach is that we can simultaneously identify off-target inhibitors, and can in secondary assays (and potentially in primary screens in future) identify *where* in the ETC identified inhibitors target (Discussion, Lines 493-499, Lines 513-521). We do not claim that we are the first to screen for ETC inhibitors, but emphasise the advantages of our approaches.

8. A much stronger paper could be based on the novel findings with MMV688853. It would be very useful to identify the ETC target of this compound and further understand the difference in the inhibition in P. falciparum.

See our response to query 2 above. We now provide evidence that MMV688853 targets the Q_i site of Complex III (Figures 9 and 10).

Reviewer #1 Major issues:

9. Given that the parasite ETC Complex III has structural differences from the human homolog, it is likely that the inhibitors discovered in this study are selective for T. gondii and P. falciparum proteins. However, the authors could provide cell viability data to demonstrate this and/or reference the selection criteria for the MMV library if this was done previously. Would it be possible to speculate on molecular mechanisms that account for this selectivity?

We have performed experiments testing whether the ETC inhibitors identified in our screen can inhibit the proliferation of human foreskin fibroblast (HFF) cells. We observed that none of the compounds could inhibit HFF proliferation at the concentrations tested (up to 10 µM), with the exception of auranofin which inhibited HFF proliferation with an EC_{50} of 2,793 ± 914 nM, a 27-fold greater concentration than the EC_{50} against WT parasite proliferation. We have reported these data as well as the selectivity index relative to *T. gondii* WT parasites in Table 1, provided the dose-response curves as S2 Fig, and added a paragraph to describe these data in the results section (Lines 201-208).

10. For the intracellular proliferation assays shown in Fig 4, “vacuoles containing 1-8+ (gray tones) or abnormal (orange) parasites” were quantified. It is stated that abnormal morphology was defined as vacuoles that contained misshapen parasites, but how this assessment was made is not clarified in the legend or methods. Was this done by eye or based on ellipticity? Could metrics be given that defined “abnormal” and representative images be included in the SI.

We have included representative images of parasite vacuoles in the various drug treatments for this experiment, including vacuoles that we classified as abnormal, in S6 Fig. We counted the parasites per vacuole by eye in a blinded manner and made decisions about abnormality based on parasites deviating from their usual morphology. We have added additional description to the Methods section to clarify this (Lines 885-888).

Reviewer #2 major issues:

11. The focus and the title/abstract of the manuscript are misleading – this is at best a "mini" screen and on a set of compounds that has been previously screened by many. There is no evidence for the scalability and the idea of targeting the ETC is one well visited in the literature and by many previous screening campaigns. I would redo the paper with a focus on the dual action compound discovered.

See response to comment 7.

12. I would suggest putting much of the data reported in the manuscript in supplemental materials.

We disagree and don't see any rationale for this. We include all the data that contribute to the main conclusions from the study in the figures and tables. Additionally, we have included numerous supplementary figures that provide readers with clarity on how we performed or quantified certain experiments (e.g. dose response curves that led to EC_{50} calculations, flow cytometry gating strategies, representative images from microscopy quantifications, traces from enzymatic assays, and quantifications of data that were presented qualitatively in the main body of the manuscript). We believe our manuscript is as concise as possible given the broad scope of the experiments, and that the reader will follow the story better if we include the initial screening and validation data in the main body of the manuscript rather than buried in the supplementary.

13. I think it is valuable to show validation of the Seahorse assay, but I would do it in the context of characterizing MMV688853 mechanism of action. A much stronger paper could be based on the novel findings with MMV688853.

See our response to queries 2 and 8. While we agree that the MMV688853 data are interesting and novel, we believe that other aspects of the paper (characterising the off-target effects of auranofin, identifying MMV024397 as a novel ETC inhibitor chemotype, demonstrating that ATV^R and ELQ^R mutants aren't cross resistant to some of these compounds) are also interesting and worth highlighting in the manuscript.

14. It would be very useful to identify the ETC target of this compound and further understand the difference in the inhibition in P. falciparum. This will require additional experiments and a more detailed analysis of this compound mechanism of action for P. falciparum - similar to that which was already presented for T. gondii. SAR around

MMV688853 would greatly strengthen the paper and provide insight into the dual inhibition.

See our response to query 2.

Reviewer #3: Major issues:
15. The main concern of the protocol using the Seahorse XFe96 assay is that only 2 plates were run for the assay of 400 compounds and 3 compounds were tested sequentially in the same well without washing or removing the previous drug. Therefore, it seems that there could be potential for false positive hits. This could present problems when scaling up to do HTP screening if that is the future direction of this screen. It would be important to report Z' score for the plates tested to show the robustness of the assay. This is relevant considering the need to use Cell Tak which most likely changes the physiology of the cells.

We specify in the methods section that we retested compounds from wells injected following the injection of 'hit' compounds (Lines 710-713). Like any screening approach, follow-up studies are necessary to validate 'hit' candidates – false positives will be picked up in these subsequent studies. As suggested by the reviewer, we calculated a Z' factor for the assay and report this in the methods section (Lines 716-720). In terms of the effects of Cell-Tak, we cannot rule out that Cell-Tak is having some effects on parasite physiology (although we also have no evidence to suggest that it does). We have previously tested the assays we use in our manuscript on strains in which we can genetically impair specific protein complexes in the ETC. We find, as predicted, that genetic impairment of Complex III but not Complex IV, can be rescued through the addition of TMPD, and that both malate and glycerol 3-phosphate-dependent OCR is impaired in both Complex III and IV mutants (*e.g.* Hayward et al., 2021, PMID: 33524071; Hayward et al., 2022, PMID: 35118179). These genetic approaches indicate that our screens can detect changes to parasite physiology associated with selective impairment of components of the ETC. In more recent unpublished studies, we have used poly-D-lysine (a much cheaper alternative to Cell-Tak) to coat Seahorse plates and the data we obtain are essentially indistinguishable from when plates are coated with Cell-Tak (example data below).

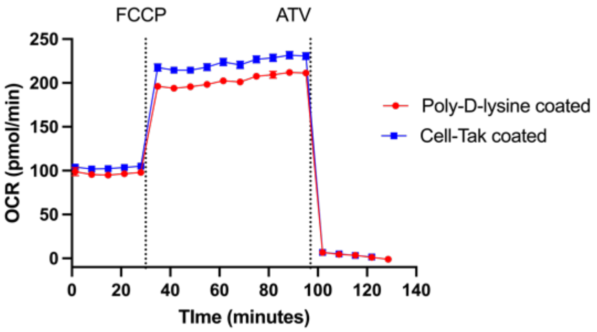

16. Fig 5: The presentation of the data could be clearer. As presented, it is not clear the significance of the inhibition by each drug so bar graphs showing the difference between the OCR before and after adding drugs for each one should be included.

Statistics should also be shown. Also, in the figure the dotted lines appear to be shifted as the change in slope occurs prior to the addition of the reagent. Fig. 6 has similar issues and also the shift of the dotted lines.

We chose to include traces from a single experiment, representative of 3 independent experiments, in Fig 5 because we felt that this gave readers the best visual appreciation of the data and the changes that were occurring in each experimental condition. We have now quantified and statistically analysed the extent of malate- or glycerol 3-phosphate-dependent OCR inhibition by each compound, and the extent of TMPD rescue, across the independent experiments. We include these new analyses in S7 Fig. These analyses support our conclusions that all tested compounds inhibit both malate- and glycerol 3-phosphate-dependent OCR, and that all compounds except auranofin can be rescued by the addition of TMPD, indicating that these compounds target Complex III.

Regarding the dotted lines, these accurately represent the time at which the compounds were injected. Regarding the slope of the lines, the measurement of OCR is not continuous, but instead taken at the defined timepoints represented by the data points in the graph. The lines connecting these data points are therefore meant to aid interpretation of the patterns in the data (we note that this is a standard way in which Seahorse data are plotted: *e.g.* Salabei et al, 2014, PMID 24457333). We now clarify all this in the figure legends for Figs 5 and 6: "Dotted lines represent the time points of each injection, and data points for each condition have been connected by lines to aid interpretation." (Lines 1250-1252; Lines 1268-1269)

17. Is there a reason for not using succinate as substrate? Previous work with Toxoplasma oxygen consumption showed that it is a better substrate than malate and much better than G3P (see table 1 of JBC273: 31040). I think that succinate is a more direct substrate. I suggest to test it at least for the experiments with Toxoplasma

We have tested succinate as a substrate in preliminary assays, but find that succinate is unable to stimulate OCR. This could be because the *T. gondii* mitochondrion lacks a succinate carrier (*i.e.* exogenously supplied succinate cannot gain access to the mitochondrial matrix). For the purposes of our experiment, we wanted to distinguish between compounds that inhibited Complex III and those that inhibited one of the dehydrogenases/oxidoreductases that feed electrons into the ETC. An inhibitor of a TCA cycle enzyme such as succinate dehydrogenase or malate:qunione oxidoreductase (which replaces malate dehydrogenase in the TCA cycle of apicomplexan parasites; PMIDs 35398098 and 31381948) could lead to general impairment of electron flow from the TCA cycle into the ETC (*i.e.* it is conceivable that an SDH inhibitor would lead to impairment of malate-dependent OCR since it would block the TCA cycle). We therefore wanted to use both a TCA cycle-*dependent* substate (malate) and a TCA-cycle-*independent* substrate (G3P) in our assays. We describe the rationale for this approach in Lines 349-358). Additionally, we would point out that malate:quinone oxidoreductase directly feeds electrons from malate to coenzyme Q and therefore malate, like succinate, is a direct ETC substrate in these parasites.

18. It is not clear why the authors use such high concentrations of inhibitors for the assays with permeabilized parasites when growth or oxygen consumption rates of intact parasites are inhibited at 50% at much lower concentrations. For some of the compounds like Azoxystrobin which has a growth inhibitory concentration of 310 nM the author uses 80 μM for the in vitro assay. These results may be indicating that other enzymes are being targeted in the parasites and the drugs are not specific.

In the permeabilized Seahorse experiments and the Complex III activity assay, the aim is to ascertain the ETC target of each compound. We therefore chose a single, high concentration of each compound that we would expect to almost fully inhibit OCR based on our intact Seahorse experiments (Table 3; S3 Fig; Lines 354-356). Using lower doses would not address the question we set out to test in these experiments as we would not achieve the amount of inhibition needed to, for instance, test whether TMPD can rescue OCR after inhibition.

19. One way to show that the drugs are specifically inhibiting the ETC is to show a correlation between growth inhibition to OCR inhibition. It will become clear if there is or there is no correlation. From the presented data it is hard to predict but for example buparvaquone inhibits growth with a 0.7 nM EC50 and the OCR 50% inhibition is 1.18 micromolar. Atovaquone needs 10 nM for inhibiting growth at 50% while only needs 180 nM to inhibit OCR. It appears as if buparvaquone would be inhibiting other targets in the parasites. More confusion is added using buparvaquone at 5 micromolar for the assays with permeabilized parasites and the assay of complex III enzymatic activity. The case of Azoxystrobin is also puzzling as it inhibits 50% growth at 310 nM, OCR at 7 micromolar and for the in vitro assays the authors use 80 micromolar. I suggest to test lower concentrations of these compounds for the experiments with permeabilized cells.

See the response to comment 1. We now include a graph that compares the proliferation and OCR EC_{50} values of the inhibitors identified in our screen (S3J Fig). We find that there is a general correlation between EC_{50} values, with the most potent compounds against OCR also being the most potent proliferation inhibitors. As the reviewer points out, buparvaquone, which appears to be a particularly potent inhibitor of proliferation relative to its ability to inhibit OCR, is a curious outlier here. Given that a single point mutation in cytochrome *b* from Complex III imparts >200-fold resistance to the effects of buparvaquone on growth inhibition (Table 1), and that *Plasmodium* parasite proliferation is rescued from buparvaquone inhibition when expressing yDHODH, we think it is unlikely that buparvaquone has an “off-target” effect or second target in *T. gondii*. It is conceivable that, in comparison to the other inhibitors we identify, buparvaquone has a greater ability to access the parasite mitochondrion in infected host cells, or may be less likely to degrade or be sequestered to other sites in the culture conditions of the proliferation assays. We note that EC_{50} values against OCR are higher across the board than against proliferation, likely due to the different time scales across which we conduct these assays and the different biological processes we are measuring (we now describe these possibilities in the Results, Lines 256-268).

In terms of why we used high concentrations of inhibitors in certain experiments, see our response to comment 18.

20. The inhibition of Plasmodium OCR is stronger for most compounds, but authors use 10 micromolar for all the in vitro assays.

We did in fact perform the *Plasmodium* OCR measurements at a range of concentrations, and used this to calculate the EC_{50} concentration for OCR inhibition (Table 3 and S9 Fig). We chose to use a high concentration of each drug for the experiments presented in Fig 6 for the reasons outlined in our response to comment 18. As the reviewer notes, *P. falciparum* EC_{50} OCR values are lower than in *T. gondii* (Table 3). We are not sure why these difference exists. It is conceivable that this reflects differences in the ETC between the two parasites (*e.g.* structural differences in Complex III), although it is notable that we calculated *T. gondii* EC_{50} values in parasites with an intact plasma membrane and *P. falciparum* EC_{50} values permeabilised parasites in *P. falciparum*. It is therefore conceivable that the lower EC_{50} values in *P. falciparum* represent greater drug accessibility in the permeabilised parasites. We have made note of these differences between OCR EC_{50} values between *P. falciparum* and *T. gondii* and the way they were measured in the main text (Lines 392-395) and in the Table 3 legend (Lines 251-252).

21. Auranofin which the authors claim may not be targeting the mitochondria ETC shows a 50% inhibition of the OCR at 2.48 micromolar which is similar to the MMV024397 which the authors claim does inhibit the ETC. This needs to be addressed in the discussion.

The reviewer is correct that we can't rule out the possibility that auranofin directly targets the ETC in addition to the effects that it is imparting on parasite viability, although its rapid impact on *T. gondii* viability indicates that it is acting in a different manner to the other compounds identified in our screen (Fig 3). We have modified the discussion to now read: "Although we cannot entirely rule out the possibility that auranofin has a direct effect on the ETC, taken together our data suggest that auranofin kills apicomplexan parasites via an ETC-independent process." (Lines 503-505)

Reviewer #1: Minor issues:

22. It would be helpful to the readers to introduce the Seahorse XFe96 flux analyzer as this is not a common instrument.

We have added the following text to open the last paragraph of the introduction: "We have recently established some versatile approaches to probe ETC function in apicomplexan parasites using a Seahorse XFe96 flux analyzer. These approaches enable us to simultaneously determine the parasite mitochondrial oxygen consumption rate (OCR), a measure of ETC activity, and the parasite extracellular acidification rate (ECAR), a proxy for parasite metabolic activity and viability [refs]. We have further adapted these assays to enable us to diagnose where in the ETC specific defects arise [refs]." (Lines 130-135)

23. Given the complex lifecycles of T. gondii and P. falciparum, the authors should refer to specific cell types and stages of the parasite lifecycles rather than saying 'host cells' for in vitro experiments.

We have modified the text where we first describe the compound screen in the results section as follows (changes underlined):

"To investigate this, we screened the MMV 'Pathogen Box' compound library (a library of 'diverse, drug-like molecules active against neglected diseases') for inhibitors of parasite mitochondrial OCR in the disease-causing tachyzoite stage of *T. gondii* parasites." (Lines 162-164).

We already specify that we are conducting the experiments on asexual blood stages of *P. falciparum* in the Results section (Lines 210-212), but we have added additional text to the methods to reemphasise this, and also to point out that we are using human erythrocytes as host cells: "Asexual blood stages of 3D7 strain *P. falciparum* parasites were maintained in synchronous continuous culture using O^+ human erythrocytes in Roswell Park Memorial Institute (RPMI)-1640 medium …" (Lines 660-662).

We specify in the Methods section that "Tachyzoite-stage *T. gondii* parasites were cultured in human foreskin fibroblasts (HFF)…" (Line 611).

24. While the authors determine that several of their compounds do not cross-react with atovaquone resistant parasites with mutations at the cytochrome b protein, this does not confirm that the parasites would not develop drug resistance with a different mutation. While it is mentioned in the methods that the authors selected for clonal populations for a single atovaquone mutation, it should be made clear in the main text that this study was performed with only one of the known mutation sites in cytochrome b. Performing assays with additional cytochrome b mutations as cited in the discussions or binding studies would provide more confidence that these compounds have novel modes of action outside of the atovaquone binding site.

See our response to comment 6. In addition to the original data on the Q_o site mutants of *T. gondii* and *P. falciparum*, the revised manuscript now incorporates the characterisation of a Q_i site *T. gondii* mutant (see additional Figures 9 and 10, and additional data in Tables 1 and 3). We have modified the text to describe these new data. As noted in our response to comment 6, our study was not intended to be a comprehensive analysis of resistance-conferring Q_o and Q_i site mutations, and we indicate this in the discussion: "…we note that several other Q_o and Q_i site mutations can confer atovaquone or ELQ resistance [12, 19, 20, 56], and as such further, more comprehensive studies should test whether these compounds are effective against other ATV^R and ELQ^R strains. (Lines 554-557).

Reviewer #3: Minor issues:

25. Table 1 & 2 (and throughout): IC50 is usually used for inhibition of a specific target like inhibition of an isolated enzyme. For growth assays EC50 is more appropriate. For the inhibition of oxygen consumption, it would be appropriate to use O2-EC50. It is not clear if Table 2 is showing the inhibition of OCR in intact parasites or permeabilized parasites. The protocol used is not clarified in the Table legend.

We have modified the text to use EC_{50} throughout. We also now specify in the Table 3 legend that "*T. gondii* experiments were conducted on intact parasites, and *P. falciparum* experiments measured malate-dependent OCR in digitonin permeabilized parasites." (Lines 251-252; see also our response to Comment 20)

26. The method section is missing the statistical analyses of the data and also clarification of the number of experiments and replicates for all assays.

We describe the statistical analyses that we undertook in the Table and Figure legends of the original manuscript. We have now also included a "Data Analysis" subsection in the methods in which we re-describe the statistical analyses that we performed (Lines 907-922).

27. For the flow cytometry methods, it says that data are exported for further analysis using FlowJo but there is no description of what analysis was done.

We now include a supplementary figure that describes the gating strategy (S5 Fig). We have expanded the methods section to provide a more detailed description of the gating (Lines 821-826).

28. Fig 2: Statistical analysis of WT vs yDHODH parasites is missing

We were unable to determine EC_{50} values for most compounds in the yDHODH line (with the exceptions of auranofin and chloroquine), precluding a statistical analysis comparing EC_{50} values in WT vs yDHODH parasites for most compounds. Given that the (lack of) differences in the auranofin and chloroquine data are clear from the graphs, we've chosen not to include statistical analyses of these.

29. Fig 3: Could use a 2-way Anova and compare the drug treated vs the control.

We have now also plotted the OCR and ECAR data as individual column graphs (S4 Fig), and, as suggested by the reviewer, we have undertaken a statistical analysis of the various inhibitors compared to a no-drug control. Given that Fig 3 enables a visual comparison of OCR and ECAR on the same graph, we have chosen to keep this as the main figure in the text.

30. Fig 4C: The colors of the no drug bars are difficult to differentiate between 4, 8, and 8+ parasites per vacuole.

We have adjusted the grey tones in Fig 4C to provide greater contrast.
