# Supplementary material for: A screen of drug-like molecules identifies chemically diverse electron transport chain inhibitors in apicomplexan parasites
Source: PLoS Pathog. 2023 Jul 20;19(7):e1011517. doi: 10.1371/journal.ppat.1011517 (PMC10403144; doi:10.1371/journal.ppat.1011517)
Supplement: Attachment — Submitted filename: Response_to_reviewers.pdf [file ppat.1011517.s012.pdf]

We thank the editor and reviewers for their comments and feedback on the previous version of our manuscript. Below, we address each point raised by the editor and Reviewer 3, with the editor/reviewer text in black and our response in blue. We have numbered each of the queries to enable cross-referencing in our responses. We have highlighted changes to the manuscript text in the marked-up manuscript accompanying our re-submission (changes in red text).

Editor comments:

1. The risk of interference caused by addition of the second and third drug should be stated as a potential weakness. While this could lead to 'overlooking' potentially interesting candidates, it does not change the fact that the 7 candidates identified were individually tested and thoroughly investigated for their target and MoA.

See our response to Reviewer 3, comments 4 and 6. We have added additional text to the discussion to note these weaknesses and how they can be overcome (Lines 527-532)

2. The limitation in regard of scalability of the screen is already addressed. It identifies compounds with a certain MoA and should not be compared to screens that 'just' identify an EC50 (measurement of replication/fitness).

We agree with the editor on this point. See our response to Reviewer 3, comment 4.

3. ECAR value is shown as a reference in the Figure S4. The authors should deposit the data from all screens on a data depository.

We have included a new supplementary table (S1 Table) that includes all the 'raw' O_2 and pH readings from all timepoints and wells of the screening plates (Tabs 4A and 4B), the plate mapping (Tabs 1A and 1B), the calculated O_2 consumption and extracellular acidification rates for each well and each measurement timepoint (Tabs 2A and 2B), data from the O_2 and pH calibrations in each plate (Tabs 5A and 5B), and summary data that indicates the mitochondrial OCR values pre- and post-compound injection and the percent inhibition of mitochondrial OCR for each MMV compound analysed in our study (Tab 3). The percent inhibition values from these summary data were used in generating Fig 1. In compiling these data, we noticed that Fig 1 of the previous submission was an older version in which we did not account for non-mitochondrial OCR in each of the plates. We have therefore also updated Fig 1 with the correct figure that includes percent inhibition values calculated to take into account the non-mitochondrial OCR. We have added additional text to the methods section of the manuscript to more clearly describe the screening experiments and data treatments (modifications to Lines 726-739), and provide a description of the contents of S1 Table in the accompanying legend (Lines 1335-1372).

Reviewer #3 comments:

4. The assay has limitations. The Z' score is not great and can barely distinguish a hit from a non-hit. A good Z' factor is usually >0.5. The main reason could be the addition of a second and a third drug to the same well. The second and the third addition are tested under different conditions. What about if there is antagonism between drugs? Or synergy? What about if a drug causes detachment of the parasites? This could also result in false negatives or result in variability. This is a problem with the assay, and the authors would need to acknowledge it. I think that

the screen is a weakness of the work and according to the data presented it may not be easily scalable.

The sorts of limitations that the reviewer mentions are inherent in a screen like ours. It is for this reason that we retested each of the candidate 'hit' compounds in secondary assays (e.g. Fig 3, Table 3). We have added a couple of sentences to the discussion to highlight these limitations and how they can be overcome.

Lines 527-532. "A limitation of the screen is that we inject multiple compounds into the same well, which may mask inhibitors that are injected after 'hit' compounds, or lead to additive or confounding effects in compounds that we identify as hits. Follow-up tests of compounds injected following hit compounds can determine whether these too inhibit OCR, and secondary screens that test hits in isolation are important to further validate those compounds."

We disagree that our screen is a weakness of the study – it formed the starting point from which we were able to identify six on-target ETC inhibitors in a library of 400 compounds. All screens have strengths and weaknesses, and it is almost always necessary to follow up 'hits' in secondary assays.

5. The authors mentioned that the main advantage of the screen is the simultaneous measurement of OCR and extracellular acidification rate (ECAR) but they do not show this second part. Figure 1 only shows the OCR and they only measured ECAR for the selected hits. Is it possible to measure ECAR at the same time as OCR for the whole plate?

Unfortunately, some of the wells in the Seahorse plates failed the pH probe calibration on the days that we undertook the screen (S1 Table; Tab 5A). We therefore cannot trust the ECAR data obtained for all wells on that day and we have chosen not to report them for the assay (although the ECAR values for each compound are available in the data presented in S1 Table). We describe this in the S1 Table legend. We note that we tested ECAR value for each of the hit compounds in Fig 3A and S4B Fig (which provided the initial clues that one of the hit compounds, auranofin, had 'off-target' effects).

6. The rest of the work is good as the authors discovered new ETC inhibitors that are also effective against plasmodium. I would re-focus the work on the characterization of the mechanism of inhibition of these new drugs instead of focusing on the screening which is a weakness.

See our response to comment 4. We disagree with the reviewer that we should refocus the work – it is necessary for us to describe the screen first before we characterise the molecular targets of the inhibitors identified from that screen. Our screen was able to identify six on-target inhibitors of the ETC in these parasites (so by that measure was successful), and we go on to define the molecular target of each in subsequent assays *i.e.* demonstrating that each of the on-target compounds inhibits Complex III (Fig 5), with MMV688853 likely inhibiting the Q_i site of Complex III (Fig 10).

7. The EC50 for growth and for Oxygen rate could be differentiated by using O2-EC50. This distinction will add clarity to the description of the results. As presented it is not clear to which EC50 they are referring.

To distinguish them from proliferation EC_{50} values, we now refer to the OCR EC_{50} values as "EC_{50}^{OCR}" throughout the manuscript and in relevant figures (Fig 4D and Fig 10) and tables (Table 3 and 4).

8. In the description of Figure 2 the letters are not mentioned.

In the results section, we describe the data in Figure 2 collectively rather than referring to individual compounds. We have therefore not referred to each letter in the figure separately.

9. Is there a reason why the EC50 for the OCR measurements between ATOs vs ATOr lines in Table 3 was not determined? May be re-design the table so that there are not so many NDs?

We were interested in determining the OCR EC_{50} values for MMV688853 in atovaquone-resistant and ELQ-300-resistant parasites since the proliferation assays to test for cross-resistance in these was complicated by MMV688853 having dual targets (as we explain in the results – Lines 471-476). To address the reviewers point, we have split Table 3 into two tables. The new Table 3 includes OCR EC_{50} values in wild type *T. gondii* and *P. falciparum* parasites for all the hit compounds. The new Table 4 includes OCR EC_{50} values for MMV688853 (and atovaquone and ELQ-300 as controls) in atovaquone-resistant and ELQ-300-resistant *T. gondii* parasite strains and the corresponding parental strains.

10. Figure S7C: a positive control with antimycin a and a negative control with a drug that inhibits downstream to Cyt c would be appropriate as the figure shows all drugs that rescue.

These data provide a quantitative analysis of the OCR traces we present in Figure 5. We use atovaquone (not antimycin A) as a 'positive' control (i.e. a known inhibitor of Complex III in the ETC than can be rescued by TMPD addition). In Figure 5, we show that addition of an inhibitor downstream of CytC (the Complex IV inhibitor sodium azide) does indeed inhibit OCR post TMPD addition.

11. Concerning the response to number 20 in the response. Using permeabilized plasmodium may give false positives for drugs that are not able to enter the parasite. It would not be comparable to the OCR measurements of T. gondii.

The reviewer is referring to our observation that the candidate inhibitors were, in general, more potent at inhibiting OCR in *P. falciparum* than in *T. gondii*. We pointed out in response 20 that the way we measure OCR EC_{50} values was different between the two organisms – we determine OCR EC_{50} in intact *T. gondii* parasites and permeabilised *P. falciparum*, which could explain the differences. So the reviewer is correct that the OCR EC_{50} values are not necessarily comparable between *T. gondii* and *P. falciparum*, and this is exactly the point we are making. In both cases, however, we are measuring OCR, so the reviewer is not correct that the *P. falciparum* data may be giving false positives.

12. The discussion needs some work as it is repetitive of the results. It even mentions the figures again.

We refer to figures in the discussion to bring the reader's attention to the particular data that we are discussing. A typical structure for our discussion paragraphs is to highlight interesting aspects of our data, compare this to what it known in the literature, and, where

appropriate, to consider alternative explanations for the data and/or highlight limitations in our understanding and/or highlight future directions that would be worth pursuing.
